# Sex Disparity in the Effect of Obesity in Hospitalized COVID-19 Patients: A Retrospective Cohort Study From the New York City Metropolitan Area

**DOI:** 10.7759/cureus.15235

**Published:** 2021-05-25

**Authors:** Ashutossh Naaraayan, Abhishek Nimkar, Sushil Pant, Amrah Hasan, Momcilo Durdevic, Henrik Elenius, Corina Nava Suarez, Stephen Jesmajian

**Affiliations:** 1 Internal Medicine, Montefiore New Rochelle Hospital, Albert Einstein College of Medicine, New Rochelle, USA

**Keywords:** covid-19, sex differences, disease mortality, body mass index: bmi, age and ageing

## Abstract

Introduction: Obesity has been recognized as a risk factor for poor outcomes in coronavirus disease 2019 (COVID-19) illness. We analyzed the impact of patient characteristics including obesity on hospital mortality and specifically analyzed the effect of obesity by body mass index (BMI) class and by sex.

Methods: This retrospective case series included adult patients consecutively hospitalized with confirmed COVID-19 illness between March 12, 2020 and May 13, 2020, at a teaching hospital in the New York City (NYC) metropolitan area. Data were manually extracted from electronic health records by the authors and included demographics, comorbidities, laboratory parameters, and outcomes (hospital mortality or discharge). We used univariable and multivariable logistic regression methods to explore the risk factors associated with in-hospital death.

Results: Some 348 patients were included in this study, of whom 207 were discharged and 141 died in the hospital. Multivariable regression showed increasing odds of in-hospital death with older age and excess weight. Interestingly obesity increased mortality in women [odds ratio (OR) 4.4, confidence interval (CI) (1.4-13.5) p=0.01] but not among men [OR 1.4, CI (0.5-3.6) p=0.5]. Among women, the effect of excess weight on mortality was seen in a “dose-effect” fashion, with increasingly higher odds of mortality from progressively worsening obesity (OR ranging between 2.7 and 6.9). Out of all the comorbidities, only obesity positively correlated with peak levels of C-reactive protein (CRP).

Conclusion: Advancing age is a risk factor for in-hospital death during COVID-19 illness. Obese women could be at a higher risk for mortality due to COVID-19 and should take extra precautions to prevent contamination by social distancing and other measures. Immunomodulators may be more effective in obese women affected by COVID-19. Further studies are needed to help elucidate this association.

## Introduction

At the time of writing, 141 million cases of coronavirus disease 2019 (COVID-19) caused by severe acute respiratory syndrome coronavirus 2 (SARS-CoV-2) have been described worldwide, resulting in three million deaths [1]. Initially reported as a local outbreak in Wuhan, China on December 31, 2019, this illness caused by a novel coronavirus rapidly transitioned to become a global threat and was declared a pandemic by the World Health Organization on March 11, 2020. To date, laboratory-confirmed cases have been documented from 192 countries across six continents. Through March and April 2020, the New York City (NYC) metropolitan region was the epicenter for COVID-19 [2]. A concerning number of cases and deaths are now being reported globally from India, Brazil, and other nations.

Although COVID-19 illness mainly manifests as fever, cough, myalgia and fatigue, illness severity can run the spectrum from asymptomatic or paucisymptomatic infection (80% of patients) to severe infection requiring hospitalization, at times with life-threatening sepsis, multi-organ failure, and death [3]. It is crucial to identify the demographic and comorbid patient factors that contribute to severe illness causing hospitalization and death. Here, we present data analyzing patient risks for mortality during COVID-19 hospitalizations, from one of the earliest epicenters in the United States [2]. Obesity has emerged as a major risk factor for mortality in COVID-19 [4]. We specifically investigated the impact of different obesity classes on mortality in hospitalized patients and the impact of obesity in patients with COVID-19 by sex.

## Materials and methods

Study design and participants

This retrospective case series included adult (>18 years old) patients consecutively hospitalized with confirmed COVID-19 illness between March 12, 2020 and May 13, 2020, at a 242-bed teaching community hospital in the NYC metropolitan area. The hospital serves approximately 250,000 people in southern Westchester County, New York. Cases were confirmed through positive results for the SARS-CoV-2 virus by reverse-transcriptase-polymerase-chain-reaction testing of a nasopharyngeal swab specimen.

Outcome data were followed up until May 22, 2020. Of the 365 patients admitted during the study period, those who were transferred to another facility for tertiary level care (n=5), those who left against medical advice (n=2) were excluded from the analysis. We did not have a good representative sample of underweight patients (n=10) and thus excluded these patients from analysis. After the above-mentioned exclusions, the final analysis included 348 patients.

Data collection

Data were manually extracted from electronic health records by the authors and included demographics, comorbid conditions, certain laboratory parameters, and outcomes (hospital mortality or discharge). Laboratory data for the highest values of peripheral blood C-reactive protein (CRP) (peak-CRP) and D-dimer (peak-D-dimer) levels during hospitalization were recorded. Three authors (AN, AN, and SP) independently reviewed the data for accuracy.

Definition of patient characteristics

Comorbidities derived from the patients or nursing home (NH) transfer forms were extracted from physician documentation on the electronic health records. The race was classified into one of the five categories: Caucasian, African-American, Hispanic, Asian or Pacific-Islander, and others. Cardiac disease was defined as chronic heart conditions including coronary artery disease, previous myocardial infarction, cardiac arrhythmias, congestive heart failure, presence of pacemaker or defibrillator device, and previous coronary artery bypass grafting or percutaneous coronary intervention. Renal disease was defined as the presence of chronic kidney disease with or without the need for dialysis. Malignancy was defined as the presence of active or a history of previous malignancy. Smoking was defined as an active or remote history of smoking. Body mass index (BMI) was used to classify patients into normal weight (BMI 18.5-24.9 kg/m2), overweight (BMI 25-29.9 kg/m2), obese (BMI >=30 kg/m2), and underweight (BMI <18.5 kg/m2) based on the classification by the World Health Organization. Obesity was further classified into Class I, Class II, and Class III based on WHO definitions.

Outcome measures and statistical analysis

The study was done according to STROBE guidelines for observational studies [5]. We computed mean, standard deviation, median with inter quartile range (IQR), frequency, and percentages as our descriptive variables. Differences in mean and percentage were assessed using the t-test and chi-squared test respectively. Differences in the median were calculated using the Mann-Whitney test.

Our main outcome was in-hospital mortality, hereby referred to as mortality. Length of hospital stay was recorded as a secondary outcome. Peak-CRP and peak-D-dimer levels were correlated with baseline patient characteristics, and analyzed as secondary outcomes.

To explore the risks associated with mortality, logistic regression analysis by univariate and age-adjusted models were performed for patient characteristics. Subsequently, to determine the independent association between these characteristics and mortality, multivariable logistic regression models were used to estimate odds ratios (ORs) adjusted for clinical covariates. Multivariable analysis was performed using the least absolute shrinkage and selection operator (Lasso) inference, a standard model building approach [6]. Lasso is most useful for sparse high-dimensional models such as ours. Cross-fit partialing-out, also known as double machine learning (DML), was used for estimation as this inference method is robust in identifying true variables among the specified potential control variable [7]. Stata version 16.0 was used for analysis (Stata Corp, Houston, TX). Age, sex, hypertension, diabetes, cardiac disease, and BMI class were always included as controls in this model by “forcing” Lasso unless one of these was the variable of interest. Lasso selected variables in the model from the following covariates [race, NH residency status, chronic obstructive pulmonary disease (COPD), renal disease, malignancy, and smoking status]. When one of these covariates was the variable of interest, it was not specified to Lasso for model selection. Two-tailed p<0.05 was considered statistically significant.

Inflammatory over-reaction and endothelial damage leading to vasculitis and thrombosis have been described as prominent manifestations in critically ill COVID-19 patients. We collected information on markers of inflammation (peak-CRP) and thrombosis (peak-D-dimer). Data for peak-CRP were available in 164/348 patients and for peak-D-dimer in 225/348 patients. We did not specify peak-CRP and peak-D-dimer as covariates in the Lasso inferential models as they are not the patients’ baseline characteristics (demographics and comorbidities). A similar approach has been taken by other researchers while analyzing outcomes data in COVID-19 [8-9]. In addition, from a pathophysiologic standpoint, they are considered a consequence of the illness and perhaps a measure of disease severity [10]. The goal of our study was to identify high risk-comorbidities and demographic characteristics from all the baseline patient characteristics that portend a worse outcome (death). We were additionally interested in finding out the baseline characteristic that would affect the levels of peak-CRP and peak-D-dimer, essentially causing severe COVID-19. Indeed, peak-CRP and peak-D-dimer were considered an outcome in our study and identified as dependent variables. Age-adjusted and multivariable (Lasso inference) linear regression models were used to identify the effect of patient characteristics on peak-CRP and peak-D-dimer. Covariates used in the inferential model for peak-CRP and peak-D-dimer were the same as described above. Regression analysis for peak-CRP was done after excluding an outlier, serum value 1600 mg/L in a cohort with a range between 0 and 446 mg/L. We then calculated age-adjusted and multivariable model-adjusted (Lasso inference) odds of mortality using peak-CRP and peak-D-dimer as the variables of interest. In addition to baseline characteristics described above, peak-CRP and peak-D-dimer levels were additionally used as covariates for this model, unless one of them was the variable of interest. Odds of mortality for peak-D-dimer were calculated after excluding the outliers (the lowest and the highest 10% values).

Ethics

The study was approved by the departmental research review committee with a waiver of informed consent due to its retrospective design (Approval number 20.5.06).

## Results

Among the 348 patients, the median age was 71 years (IQR 59-82 years), 56.6% were men, 33.6% were white, and 36.2% presented from an NH (Table 1). Patients who died were significantly older than patients who survived (78 years vs 65 years, p<0.001). The mortality rate among admissions from NH was 53.2%, among those from the community was 33.3% and overall, 40.5%. Not surprisingly, a significantly higher proportion of people who died were from NH (48% vs 29%, p<0.001). The proportion of Caucasians among non-survivors was higher than that among survivors (40% vs 29%, p=0.03). This was because they were more likely to be from an NH (58% vs 25%, p<0.001) and were significantly older when compared to other races/ethnicities (76 years vs 67 years, p<0.001). Patients who died were more likely to have hypertension and cardiac disease when compared to patients who survived. Peak-CRP and peak-D-dimer levels were significantly higher in non-survivors compared to survivors (Table 2 and Table 3).

**Table 1:** Baseline characteristics of hospitalized COVID-19 patients. Data presented as number (percentage) except age and length of stay. COVID-19, coronavirus disease 2019; BMI, body mass index; COPD, chronic obstructive pulmonary disease; IQR, inter quartile range; LOS, length of hospital stay; SD, standard deviation; NH, nursing home.

	Entire cohort (n=348)	Survivors (n=207)	Nonsurvivors (n=141)	p value
Age, years - median (IQR)	71 (59–82)	65 (55–76)	78 (69–87)	<0.001
Sex – Male, n (%)	197 (56.6)	120 (57.9)	77 (54.6)	0.5
Race				
Caucasian	117 (33.6)	60 (28.9)	57 (40.4)	0.03
African American	123 (35.3)	77 (37.2)	46 (32.6)	0.4
Hispanic	69 (19.8)	44 (21.3)	25 (17.7)	0.4
Asian	8 (2.3)	6 (2.9)	2 (1.4)	0.4
Others	31 (8.9)	20 (9.7)	11 (7.8)0	0.6
NH status	126 (36.2)	59 (28.5)	67 (47.5)	<0.001
Comorbidities				
Hypertension	232 (66.7)	126 (60.9)	106 (75.2)	0.01
Diabetes	147 (42.2)	81 (39.1)	66 (46.8)	0.2
Cardiac diseases	113 (32.5)	57 (27.5)	56 (39.7)	0.02
COPD	48 (13.8)	27 (13.04)	21 (14.9)	0.6
Renal disease	64 (18.4)	39 (18.8)	25 (17.7)	0.8
Malignancy	67 (19.3)	35 (16.9)	32 (22.7)	0.2
Smoking status	62 (17.8)	37 (17.9)	25 (17.7)	0.9
Body habitus				
Normal BMI	104 (29.9)	64 (30.9)	40 (28.4)	0.6
Overweight	123 (35.3)	70 (33.8)	53 (37.6)	0.5
Obesity	121 (34.8)	73 (35.3)	48 (34.04)	0.8
Class – I	65 (18.7)	39 (18.8)	26 (18.4)	0.9
Class – II	33 (9.5)	20 (9.7)	13 (9.2)	0.9
Class – III	23 (6.6)	14 (6.8)	9 (6.4)	0.9
LOS (Days + SD)	8.2 + 7.3	8.1 + 7.5	8.4 + 7.1	0.7

**Table 2:** Levels of peak C-reactive protein in COVID-19. CRP, C-Reactive protein; COVID-19, coronavirus disease 2019

	All patients (n=163)	Survivors (n=91)	Non-survivors (n=72)	p value
Peak-CRP (mg/L)	176.4 + 110	136.4 + 96.6	226.9 + 105.6	< 0.001

**Table 3:** Levels of peak-D-dimer in COVID-19. IQR, inter quartile range; COVID-19, coronavirus disease 2019

Characteristic	All patients (n=225)	Survivors (n=123)	Non-survivors (n=102)	p value
Peak-D-dimer (mcg/mL), median (IQR)	1.5 (0.5–4.7)	0.7 (0.3–2.5)	2.9 (1.1–14.2)	< 0.001

On univariate analysis, those who died of COVID-19 were more likely to be older, OR 1.06 (1.04-1.08) for being older by one year, p=<0.001 (Table 4). Those who died of COVID-19 were also more likely to have hypertension and cardiac disease (Table 4). In the age-adjusted model, being overweight {OR 1.9 (1.1-3.5) p=0.03} and obese {OR 2.5 (1.3-4.6) p=0.005} correlated with mortality (Table 5). On multivariable logistic regression, patients who died had higher odds of being older {OR 1.06 (1.04-1.1) p<0.001} and belonging to BMI classes - overweight {OR 2 (1.1-3.7) p=0.03} and obesity {OR 2.5 (1.2-4.9) p=0.01}.

**Table 4:** Univariate analysis of patient characteristics with mortality. COPD, chronic obstructive pulmonary disease; BMI, body mass index; OR, odds ratio; CI, confidence interval

	OR (CI)	p value
Age (years)	1·06 (1·04–1·08)	< 0.001
Gender (Male vs Female)	0·9 (0·6–1·3)	0.5
Race (reference Caucasian)		
African American	0·6 (0·4–1·1)	0.1
Hispanic	0·6 (0·3–1·1)	0.1
Asian	0·4 (0·7–1·8)	0.2
Others	0·6 (0·3–1·3)	0.2
Comorbidities		
Hypertension	1.9 (1.2–3.1)	0.006
Diabetes	1.4 (0.9–2.1)	0.2
Cardiac diseases	1.7 (1.1–2.7)	0.02
COPD	1.2 (0.6–2.2)	0.6
Renal disease	0.9 (0.5–1.6)	0.8
Malignancy	1.4 (0.8–2.5)	0.2
Smoking status	0.9 (0.6–1·7)	0.9
Body habitus (reference normal BMI)		
Overweight	1.2· (0.7–2.1)	0.5
Obesity	1.1 (0.6–1.8)	0.9

**Table 5:** Risk factors for in-hospital mortality. BMI, body mass index; COPD, chronic obstructive pulmonary disease; CI, confidence interval; OR, odds ratio; ref, reference.

	Age-adjusted OR (CI)	p value	Multivariable OR (CI)	p value
Age	–	–	1.06 (1.04–1.1)	<0.001
Male sex (ref Female)	1.0 (0.8–1.9)	0.4	1.2 (0.8–2.02)	0.4
Race (ref Caucasian)				
African American	0.9 (0.5–1.5)	0.6	0.8 (0.5–1.5)	0.5
Hispanic	1.5 (0.7–2.9)	0.3	1.6 (0.7–3.5)	0.2
Asian	0.5 (0.1–2.8)	0.4	0.6 (0.1–3.1)	0.5
Others	0.8 (0.3–1.9)	0.6	0.8 (0.3–1.8)	0.5
Comorbidities				
Hypertension	1.3 (0.8–2.2)	0.3	1.2 (0.7–2.1)	0.6
Diabetes	1.5 (0.9–2.4)	0.1	1.3 (0.8–2.2)	0.3
Cardiac diseases	1.1 (0.6–1.7)	0.8	1.02 (0.6–1.7)	0.9
COPD	0.8 (0.4–1.6)	0.6	0.8 (0.4–1.7)	0.6
Renal disease	0.7 (0.4–1.2)	0.2	0.6 (0.3–1.1)	0.1
Malignancy	1.1 (0.6–1.9)	0.8	1.1 (0.6–1.9)	0.9
Smoking status	0.8 (0.4–1.4)	0.4	0.8 (0.4–1.4)	0.4
Body habitus				
Normal BMI (ref)	–	–	–	–
Overweight	1.9 (1.1–3.5)	0.03	2 (1.1–3.7)	0.03
Obesity	2.5 (1.3–4.6)	0.005	2.5 (1.2–4.9)	0.01
Sensitivity analysis for sex				
Among Women				
Overweight	2.6 (0.9–6.6)	0.056	2.6 (0.9–7.5)	0.08
Obesity	5.1 (1.8–14.1)	0.002	4.4 (1.4–13.5)	0.01
Among Men				
Overweight	1.5 (0.7–3.2)	0.3	1.5 (0.6–3.6)	0.3
Obesity	1.5 (0.6–3.5)	0.4	1.4 (0.5–3.6)	0.5

Analysis by sex was done to assess the effect of sex on the association of BMI classes and mortality. Among women, those who died seemed to have higher odds of being overweight, although this did not reach predetermined statistical significance (Table 5). The presence of obesity in women was significantly correlated with mortality in both the age-adjusted and multivariable model {OR 4.4 (1.4-13.5) p=0.01}. Among men, no correlation was seen between in-hospital mortality and obesity (Table 5).

**Figure 1:**
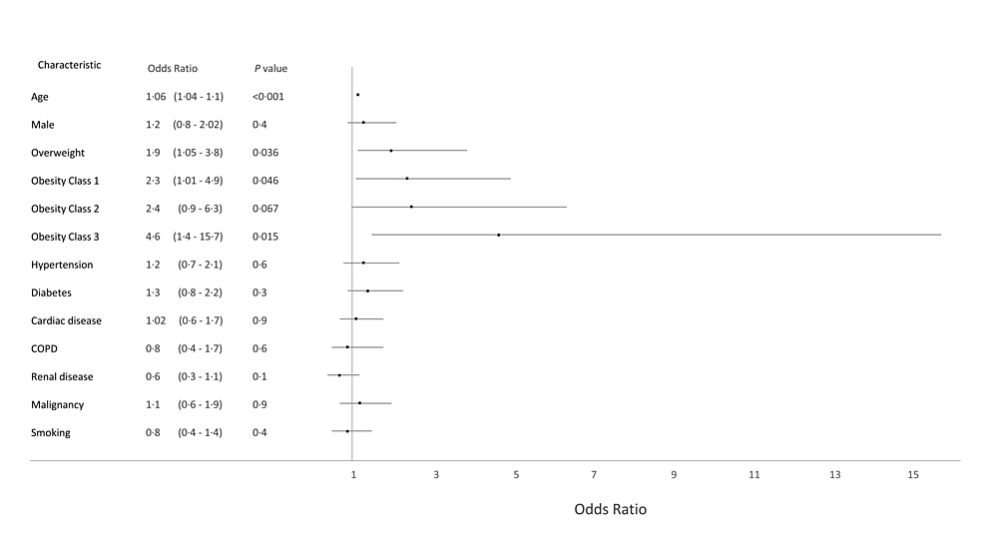
Forest plot showing adjusted odds of in-hospital mortality.

When further analyzed by the severity of obesity class, we found increasingly higher odds with progressively worsening obesity in a “dose-dependent” manner, although not always statistically significant (Table 6 and Figure 1).

**Table 6:** Risk of in-hospital death with obesity stratified by severity classes. n, number; OR, odds ratio; CI, confidence interval

	Age-adjusted OR (CI)	p value	Multivariable OR (CI)	p value
All patients				
Normal weight (n=104)	–	–	–	–
Overweight (n=123)	1.9 (1.1–3.6)	0.03	1.9 (1.05–3.8)	0.036
Obese class – 1 (n=65)	2.1 (1.02–4.3)	0.04	2.3 (1.01–4.9)	0.046
Obese class – 2 (n=33)	2.6 (1.02–6.4)	0.04	2.4 (0.9–6.3)	0.067
Obese class – 3 (n=23)	4.3 (1.5–12.6)	0.008	4.6 (1.4–15.7)	0.015
Women only				
Normal (n=52)	–	–	–	–
Overweight (n=45)	2.6 (0.9–6.8)	0.054	2.7 (0.8–7.9)	0.08
Obese class – 1 (n=29)	4.8 (1.5–15.1)	0.008	4.1 (1.2–14.1)	0.03
Obese class – 2 (n=14)	4.2 (0.9–18.4)	0.054	3.7 (0.8–17.3)	0.09
Obese class – 3 (n=11)	8.2 (1.7–41)	0.01	6.9 (1.04–46.2)	0.046
Men only				
Normal (n=52)	–	–	–	–
Overweight (n=78)	1.5 (0.7–3.3)	0.3	1.7 (0.7–4.3)	0.3
Obese class – 1 (n=36)	1.2 (0.5–3.1)	0.7	1.2 (0.4–3.7)	0.7
Obese class – 2 (n=19)	1.8 (0.6–6.1)	0.3	1.9 (0.5–7.2)	0.3
Obese class – 3 (n=12)	2.9 (0.6–12.9)	0.2	3.3 (0.7–16.9)	0.2

**Figure 2:**
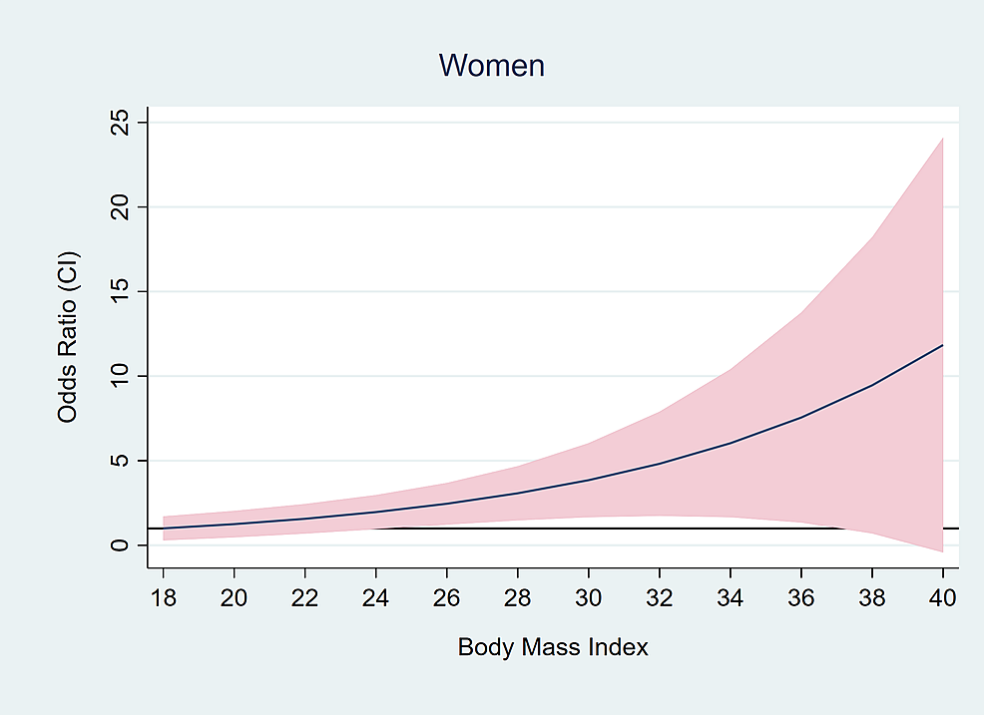
Age-adjusted odds of in-hospital mortality with increasing BMI in women. BMI, body mass index

**Figure 3:**
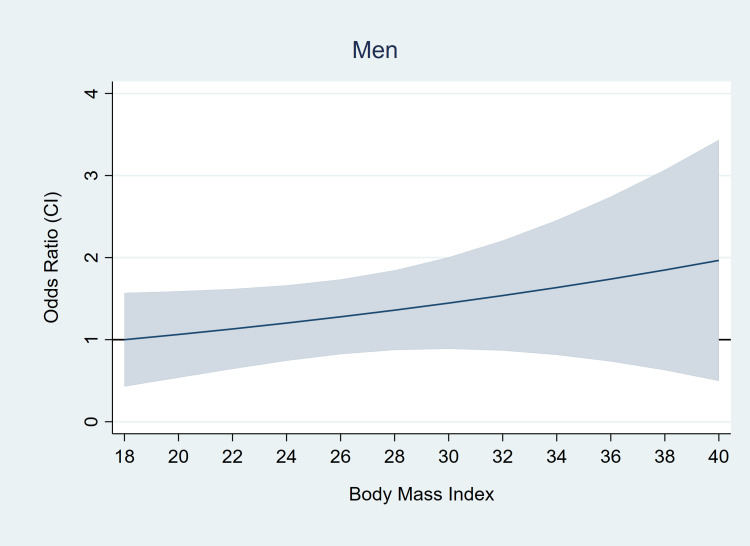
Age-adjusted odds of in-hospital mortality with increasing BMI in men. BMI, body mass index

Again, this effect was not seen in men and was primarily because of an independent correlation between excess weight and mortality in women (Table 6, Figures 2-3).

In the secondary outcome, obesity was the only patient characteristic that significantly correlated with peak-CRP levels (Table 7). Race and hypertension correlated with peak-D-dimer levels while no such association with obesity was revealed (Table 8). Peak-CRP was associated with significantly higher odds of mortality, odds ranging between 1.5 and 1.6 for every 50 mg/L increase in peak-CRP levels (p<0.001). Odds of mortality were increased with increasing peak-D-dimer levels as well (Table 9).

**Table 7:** Predictors of peak-CRP levels (measured in milligrams per liter) by linear regression analysis. COPD, chronic obstructive pulmonary disease; BMI, body mass index; ref, reference; CRP, C-reactive protein

	Coefficient (age-adjusted model)	p value	Coefficient (multivariable model)	p value
Age	–	–	1.3 (-0.1–2.7)	0.06
Male gender	16.8 (-18.8–52.3)	0.4	22.2 (-12.1–56.6)	0.2
Race (ref Caucasian)				
African American	0.4 (-41.7–42.5)	0.9	7.6 (-31.9–47.1)	0.7
Hispanic	42.2 (-7.2–91.6)	0.09	48.4 (-3.5–100.4)	0.07
Asian	-77.6 (-205–49.9)	0.2	-72.5 (-172.6–27.6)	0.2
Others	-41.3 (-104.1–21.6)	0.2	-25.5 (-79.8–28.9)	0.4
Comorbidities				
Hypertension	-3.8 (-42.3–34.8)	0.9	3.8 (-35.9–43.5)	0.9
Diabetes	-32.3 (-66.6–1.9)	0.07	-27.7 (-63.4–8)	0.1
Cardiac disease	-44.2 (-81.6– -6.8)	0.02	-37.4 (-75–0.2)	0.05
COPD	-17.3 (-70.1–35.4)	0.5	-9.8 (-61–41.5)	0.7
Renal disease	-37.8 (-83.3–7.6)	0.1	-20 (-66.7–26.7)	0.4
Malignancy	-12.6 (-57.8–32.7)	0.6	-9.8 (-53.7–34.1)	0.7
Smoking status	3.3 (-44.6–51.1)	0.9	13.1 (-34.5–60.7)	0.6
Body habitus (ref normal BMI)				
Overweight	19.2 (-24.3–62.6)	0.4	18.9 (-20.8–58.6)	0.4
Obesity	45.4 (0.4–90.5)	0.048	45.1 (5.9–84.3)	0.02

**Table 8:** Predictors of peak-D-dimer levels (measured in mcg/mL) by linear regression analysis. COPD, chronic obstructive pulmonary disease; BMI, body mass index; ref, reference

	Coefficient (age-adjusted model)	p value	Coefficient (multivariable model)	p value
Age	–	–	0.1 (0.005–0.2)	0.04
Male gender	1.3 (-12.4–3.8)	0.3	1.3 (-1.1–3.8)	0.3
Race (ref Caucasian)				
African American	5.4 (2.5–8.3)	<0.001	5.3 (2.3–8.3)	0.001
Hispanic	3.4 (-0.0004–6.8)	0.05	3.9 (0.8–6.9)	0.01
Asian	0.6 (-7.6–8.8)	0.9	0.8 (-4.2–5.9)	0.8
Others	-0.7 (-5.2–3.8)	0.8	-0.3 (-2.8–2.2)	0.8
Comorbidities				
Hypertension	2.9 (0.2–5.7)	0.03	3.5 (0.9–5.9)	0.006
Diabetes	-0.2 (-2.7–2.3)	0.9	-1.1 (-3.8–1.6)	0.4
Cardiac disease	-1.4 (-4.2–1.3)	0.3	-0.7 (-3.7–1.7)	0.5
COPD	-0.8 (-4.5–2.9)	0.7	-0.3 (-3.9–3.2)	0.9
Renal disease	-2.5 (-5.7–0.7)	0.1	-2.8 (-5.7–0.0009)	0.05
Malignancy	-1.7 (-4.9–1.5)	0.3	-1.3 (-4.3–1.8)	0.4
Smoking status	-1.9 (-5.2–1.3)	0.2	-2.1 (-5.1–0.8)	0.2
Body habitus (ref normal BMI)				
Overweight	1.5 (-1.6–4.6)	0.4	1.6 (-1.5–4.7)	0.3
Obesity	2.3 (-1.01–5.6)	0.2	1.8 (-1.3–4.9)	0.3

**Table 9:** Risk of in-hospital mortality with peak-CRP and peak-D-dimer levels. CRP, C-Reactive protein; OR, odds ratio; CI, confidence interval

Characteristic	Age-adjusted OR (CI)	p value	Multivariable model OR (CI)	p value
CRP (every 50 mg/L increase)	1.6 (1.3–1.9)	<0.001	1.5 (1.3–1.8)	<0.001
D-dimer (every 0·5 mcg/mL increase)	1.06 (1.03–1.1)	<0.001	1.05 (1.02–1.1)	<0.001

## Discussion

In this study, we present data identifying patient characteristics that portend a worse prognosis for patients hospitalized with COVID-19. Our patient cohort was diverse; with whites, blacks, and other races comprising a third of the patients each. We found significantly higher odds of mortality with increasing age. Every one-year increment in age increased the odds of death by 1.06. This is not surprising, as by now advancing age is an established risk factor for poor outcomes in COVID-19 [9, 11]. Besides age, the only other patient characteristic that impacted mortality was the presence of excess weight. Patients who died had more than two-fold odds of being overweight and obese compared to the people who survived. This effect was seen in both age-adjusted and a robust multivariable model built using a standardized approach (Lasso inference). The correlation between excess weight and mortality manifested as if it were a “dose-effect” response, i.e. in those who died progressively higher odds of belonging to a higher BMI class were observed, compared to those who survived (Figure 1). Interestingly this correlation between mortality and excess weight was seen only in women, with as much as four to five times higher odds of being afflicted by excess weight in patients who died compared to those who survived (Figure 2). The excess weight did not seem to have an effect on mortality among men, as increasing age was the only identifiable risk among men (Figure 3). It is noteworthy that in those who died, the odds for Obesity Class II failed to meet the pre-requisite statistical significance of p<0.05. We believe it is likely a result of a small sample size leading to a lack of power in detecting a significance. This is also reflected in the wide confidence intervals seen in the odds. Similarly, we believe other risk factors such as diabetes and hypertension did not meet statistical significance likely from a lack of power due to the small sample size.

In previous influenza and novel coronavirus epidemics, severe acute respiratory syndrome (SARS-CoV-1) and the Middle East respiratory syndrome (MERS), in addition to virus-induced cytopathic effects an excessive and dysregulated host immune response played a crucial role in the pathology and mortality [12-13]. This exaggerated response with supraphysiologic levels of cytokines is referred to as cytokine release syndrome or “cytokine-storm” [14-15]. Cytokine-storm plays a crucial role in COVID-19 severity and outcome [4, 14-15]. Peripheral blood levels of interleukin-6 and C-Reactive protein (CRP) correlate well with the onset and presence of cytokine storm [14]. CRP in contrast to interleukin-6 is a widely available test in most hospitals. CRP was recently shown to correlate with the extent of lung pathology and disease severity in COVID-19 patients [10].

Obesity has been increasingly identified as a risk factor for worse outcomes in COVID-19 [8-9]. Earlier data from China did not reveal this effect probably because the prevalence of obesity in China is significantly lower (6%) when compared to the United States (42%) and Europe (>20%) [9]. Data from NYC suggested that obesity might be a risk factor for ICU admission in younger patients (age <60 years) with COVID-19 [16]. Increased independent odds of mortality with obesity have been described in COVID-19 patients [9]. However, a handful of studies before us have provided analysis on odds of mortality by obesity class [17]. This association of a noncommunicable illness with outcomes of viral epidemics is not surprising or new. Obesity has been previously recognized as a risk factor for severe illness, hospitalization, and death in the 2009 influenza pandemic (H1N1) as well as during the MERS epidemic [18-19].

Our finding begs the question, what are the pathophysiologic mechanisms behind this association? Generally, individuals affected by obesity demonstrate a restrictive pattern on pulmonary function testing, have impairment of ventilation due to impedance to diaphragmatic excursions, possess reduced lung volumes, and are more likely immunosuppressed from often associated diabetes, increasing their risk of pneumonia [20].

Adipose tissue should not be considered a mere storage site of excess energy, as it plays a more complex physiologic role through endocrine and immunologic responses. Adipose tissue consists of preadipocytes, adipocytes, infiltrated B-cells, T-cells, macrophages, and neutrophils [21-22]. Adipose tissue is essentially an immunologically active organ that displays hallmarks of both innate and adaptive immune responses [21]. Obesity increases the immune cells in adipose tissue and skews their phenotype from anti-inflammatory towards more pro-inflammatory subtypes [21-22]. Thereby, obesity increases inflammation, makes the immune system more vulnerable to infections, and less responsive to vaccinations, antivirals, and antimicrobials [22]. As an example, in influenza, individuals afflicted by obesity have been shown to have high viral titers in the lung, worsened lung pathology, and increased mortality despite vaccination. By the above mechanisms, obesity causes a persistent elevation in inflammatory markers. Elevated interleukin-6, CRP, adipokines, cytokines, and interferons in obesity characterize a chronic low-grade inflammation [23]. Thus, obesity is interlinked with infections and inflammation in a manner that is far more complex than previously thought. In addition, adipocytes express angiotensin-converting enzyme 2 (ACE2) which is reportedly the mode of cellular entry for SARS-CoV-2 [24]. Therefore, a direct proinflammatory role of SARS-CoV-2 in intrapulmonary adipocytes should be considered as a mechanism of injury.

Thereby, increased adipose tissue in obese individuals could lead to an exaggerated inflammatory response and cytokine-storm in COVID-19. Indeed, obesity correlated with peak-CRP levels in our study while none of the other patient characteristics did. Unsurprisingly, peak-CRP was associated with mortality in our study, thereby supporting the pathologic role of a hyperactive inflammatory response. On the other hand, we found no association between obesity and peak-D-dimer levels. The effect of peak-D-dimer levels on mortality was significant but less pronounced than that of peak-CRP levels.

The patient’s sex did not impact the outcome of mortality in our study. In general, men experience a greater severity and prevalence of bacterial, viral, fungal, and parasitic infections than women. Women also display a more robust response to antigenic challenges such as infection and vaccination [25]. This stronger immune response in women could, however, more frequently evolve into cytokine-storm contributing to worse outcomes. During viral epidemics, the sex difference in outcomes thus depends on the balance between the beneficial anti-viral effect and the harm from an exaggerated inflammatory response. Historically, influenza epidemics have varied in their risk to the sexes, some were worse for men (1918) and others for women (2004) [26]. In previous coronavirus outbreaks (SARS-CoV-1 and MERS), men had higher mortality rates than women [13, 27-28]. Likewise, COVID-19 hospitalizations have mostly comprised men, and thus far into the pandemic, men have been identified to be at greater risk [8-9]. As mentioned earlier, a difference in mortality with sex was not seen in our study. Our patient cohort was fairly old (3/4th of patients aged >=60 years) and it is possible that the effect of advancing age on mortality overpowered that of sex. Notably, a majority of studies have reported early mortality data while a significant proportion of patients were still hospitalized. Thus, mortality rates and outcomes may change when definite outcomes are analyzed by including those patients who were still hospitalized at the time of reporting. Meanwhile, some researchers have shared findings similar to ours, with no difference in mortality rates between the two sexes [11].

Prior to our study, most researchers did not perform a sensitivity analysis looking for the effect of sex on the association between obesity and mortality in COVID-19 [8]. To the best of our knowledge, only a handful of studies have analyzed and reported sex-disparity in the effect of obesity on outcomes in COVID-19 patients [23, 29]. Interestingly, the studies found conflicting results. Researchers from California found an increased risk of obesity in men but not in women [29]. In contrast in a large cohort of patients from the UK, a higher BMI was associated with a stronger risk of COVID-19 mortality in women than in men; the women-to-men ratio of hazard ratios was 1.20 (1.00- 1.43) [23]. Similar to the study from the UK, we found a sex-based difference in the effect of obesity on mortality with a higher BMI increasing the odds of death for women but not for men. One of the reasons for this increased risk in women might be because obesity as defined by the BMI, does not correlate well with the adipose tissue mass, as BMI does not take body fat percentage into account. Women with a higher fat percentage have a higher mass of adipose tissue compared to men for the same body weight and BMI class. A greater amount of adipose tissue could mean a greater inflammatory response in the setting of COVID-19 illness leading to poor outcomes. However, our test for this hypothesis failed to show any difference in the peak-CRP levels between men and women, indicating that other forces could be at play. Previously, researchers identified a set of genes regulated by testosterone and participating in lipid biosynthesis, whose expression was negatively associated with antibody responses to influenza vaccine in men [25]. Higher testosterone levels corresponded with lower antibody response to the vaccine. This demonstrable association between sex hormones, genes involved in lipid metabolism, and altered immune response to viral antigens, gives us some insight into the rather complex associations between sex, lipid metabolism, and immunity. It is unclear if the genes involved in lipid biosynthesis had any correlation to the BMI. Further studies are needed to test these hypotheses. Our study adds to the literature supporting a higher risk with increasing BMI in women with COVID-19 as compared to men with COVID-19.

Relevance of findings

Clarifying the impact of obesity on COVID-19 severity and outcomes is of major public health importance. The last known prevalence of obesity in the United States was 42.4% in 2017-2018 and its impact was seen in all age groups. Similar to the United States, other western countries have a high prevalence of obesity as well [9]. It would be a mistake to think that developing nations are relatively protected from the impact of obesity, and thus from a potentially increased death due to COVID-19. The largest study to date demonstrated that about two-thirds of the obese population in the world was living in developing countries [30]. If our findings are generalizable, the growing spread of the illness in developing nations such as Brazil and India is of monumental concern.

Our study adds to the growing literature supporting an almost dose-effect response between excess weight and mortality [8, 17]. To the best of our knowledge, we are one of a handful of studies to report a sex disparity in this effect of excess weight on mortality [23, 29]. Due to conflicting results in the previous studies, further analyses and research is urgently needed to identify the population at risk. Given our findings and those from Lighter et al. and Peters et al., obesity could increase the risk to patient groups previously considered at low-risk, i.e. women and younger individuals [16, 23]. Unquestionably our findings are of great public health importance and need to be disseminated, corroborated, and elucidated.

In the interim, we recommend that people with obesity, especially women affected by severe obesity, should take extra precautions to avoid COVID-19 contamination by strictly enforcing social distancing and other prevention measures. Once diagnosed, patients with severe obesity should be monitored more closely. As the primary mechanism for death due to obesity is likely the immune over-reaction, these patients may benefit to a greater extent from immunomodulatory treatments aimed at the complement system or interleukin-6, which are being studied in clinical trials.

Limitations

The limitations of this study are that the study population was from a single hospital and included older patients, often from NHs, with multiple comorbidities and thus findings may not be broadly applicable to patients of different age groups. On the other hand, there are several communities spread across the United States and the world, with population characteristics similar to ours, and our experience should be directly applicable to them. Underweight patients were not well represented in our patient cohort; thus, we could not investigate outcomes in that subgroup of patients. Additionally, this is a retrospective case series and as such can only report on associations between the variables under study and cannot draw conclusions related to cause and effect. As the pandemic spread in our community and the rest of the world, and new information concerning the pathogenetic importance of cytokine storm and thrombosis became widely available, our practice patterns changed. Notably, a protocol for steroid use for cytokine-storm syndrome and anticoagulation for elevated D-dimers was adopted at our hospital and evolved as more information became available with the evolving pandemic. This most certainly was true for all the previous reports of COVID-19 as well, and in our estimation was not unique to our study.

## Conclusions

In a diverse patient cohort, advancing age and obesity were independent predictors of mortality among hospitalized COVID-19 patients. While advancing age affected both sexes similarly, obesity impacted mortality among women but not among men. The association of obesity with mortality was more powerful than that of race, and all other comorbidities including hypertension, diabetes, and cardiac diseases. Out of all the patient characteristics, only obesity independently affected the blood levels of peak-CRP in COVID-19 patients. This strengthens the hypothesis that a dysregulated and hyperactive immune response could be the central mechanism for increased risk among obese individuals. Based on our findings, immunomodulators might have higher efficacy in women affected by obesity. Further studies analyzing definite outcomes in COVID-19 are much needed. Confirmation of this increased risk with further data will help guide recommendations for the prevention, hospitalization, and management of these at-risk individuals. Our findings are of great public health importance and should be shared, corroborated, and elucidated.
